# Correction: Differential Response to α-Oxoaldehydes in Tamoxifen Resistant MCF-7 Breast Cancer Cells

**DOI:** 10.1371/journal.pone.0104322

**Published:** 2014-07-28

**Authors:** The PLOS ONE Staff

## Notice of Republication

This article was republished on July 9, 2014, due to an error in the title. The publisher apologizes for this error. Please download this article again to view the corrected title and citation. The originally published, uncorrected article and the republished, corrected article are provided here for reference.

## Supporting Information

File S1 Originally published, uncorrected article.
(pdf)

File S2 Republished, corrected article.
(pdf)

## References

[1] Nass N, Brömme H-J, Hartig R, Korkmaz S, Sel S, et al (2014) Differential Response to α-Oxoaldehydes in Tamoxifen Resistant MCF-7 Breast Cancer Cells. PLoS ONE 9(7): e101473 doi:10.1371/journal.pone.0101473 24983248 10.1371/journal.pone.0101473 PMC4077828

