# Supplementary material for: Correction: Differential Response to α-Oxoaldehydes in Tamoxifen Resistant MCF-7 Breast Cancer Cells
Source: PLoS One. 2014 Jul 28;9(7):e104322. doi: 10.1371/journal.pone.0104322 (PMC4113444; doi:10.1371/journal.pone.0104322)
Supplement: File S1 — Originally published, uncorrected article. (pdf) [file pone.0104322.s001.pdf]

Differential Response to α-Axoaldehydes in Tamoxifen Resistant MCF-7 Breast Cancer Cells

Norbert Nass[1]*, Hans-Jürgen Brömme[2], Roland Hartig[3], Sevil Korkmaz[4], Saadettin Sel[5], Frank Hirche[6], Aoife Ward[7], Andreas Simm[2], Stefan Wiemann[7], Anne E. Lykkesfeldt[8], Albert Roessner[1], Thomas Kalinski[1]

1 Department of Pathology, Otto von Guericke University Magdeburg, Magdeburg, Germany, 2 Martin-Luther-University Halle-Wittenberg, Centre for Basic Medical Research (ZMG), Halle, Germany, 3 Otto-von-Guericke-University Medical Faculty, Multidimensional Microscopy and Cellular Diagnostics, Magdeburg, Germany, 4 Department of Cardiac Surgery, University of Heidelberg, Heidelberg, Germany, 5 Department of Ophthalmology, University of Heidelberg, Heidelberg, Germany, 6 Martin-Luther-University Halle-Wittenberg, Institute of Agricultural and Nutritional Sciences, Halle/Saale, Germany, 7 German Cancer Research Center DKFZ, Division of Molecular Genome Analysis, Heidelberg, Germany, 8 Danish Cancer Society Research Center, Breast Cancer Group, Cell Death and Metabolism, Copenhagen, Denmark

Abstract

Tamoxifen is the standard adjuvant endocrine therapy for estrogen-receptor positive premenopausal breast cancer patients. However, tamoxifen resistance is frequently observed under therapy. A tamoxifen resistant cell line has been generated from the estrogen receptor positive mamma carcinoma cell line MCF-7 and was analyzed for putative differences in the aldehyde defence system and accumulation of advanced glycation end products (AGE). In comparison to wt MCF-7 cells, these tamoxifen resistant cells were more sensitive to the dicarbonyl compounds glyoxal and methylglyoxal and displayed increased caspase activity, p38-MAPK- and IκBα-phosphorylation. However, mRNA accumulation of the aldehyde- and AGE-defence enzymes glyoxalase-1 and -2 (GLO1, GLO2) as well as fructosamine-3-kinase (FN3K) was not significantly altered. Tamoxifen resistant cells contained less free sulfhydryl-groups (glutathione) suggesting that the increased sensitivity towards the dicarbonyls was due to a higher sensitivity towards reactive oxygen species which are associated with dicarbonyl stress. To further analyse, if these data are of more general importance, key experiments were replicated with tamoxifen resistant MCF-7 cell lines from two independent sources. These cell lines were also more sensitive to aldehydes, especially glyoxal, but were different in their cellular signalling responses to the aldehydes. In conclusion, glyoxalases and other aldehyde defence enzymes might represent a promising target for the therapy of tamoxifen resistant breast cancers.

Citation: Nass N, Brömme H-J, Hartig R, Korkmaz S, Sel S, et al. (2014) Differential Response to α-Axoaldehydes in Tamoxifen Resistant MCF-7 Breast Cancer Cells. PLoS ONE 9(7): e101473. doi:10.1371/journal.pone.0101473

Editor: Toshi Shioda, Massachusetts General Hospital, United States of America

Received July 11, 2013; **Accepted** June 6, 2014; **Published** July 1, 2014

Copyright: © 2014 Nass et al. This is an open-access article distributed under the terms of the Creative Commons Attribution License, which permits unrestricted use, distribution, and reproduction in any medium, provided the original author and source are credited.

Funding: The study was funded by the Danish Cancer Society Research Center, the Federal Ministry of Education and Research (BMBF – e:Bio FKZ 0316168C), German Cancer Research Center DKFZ, Otto von Guericke University Magdeburg, Martin-Luther University Halle-Wittenberg and Otto von Guericke University Magdeburg. The funders had no role in study design, data collection and analysis, decision to publish, or preparation of the manuscript.

Competing Interests: The authors have declared that no competing interests exist.

* Email: norbert.nass@med.ovgu.de

Introduction

Tamoxifen is the most commonly used anti-hormonal drug for adjuvant treatment of estrogen receptor (ER) positive premenopausal breast cancer patients. However, this is hampered by a frequently occurring development of resistance during therapy [1]. Several mechanisms have been proposed to explain the frequent occurrence of tamoxifen resistance in ER positive breast cancers [2]. Among these are increased signalling via the HER receptor system [3], altered expression of ER cofactors [4] or enhanced NFκB activity [5]. We further associated the expression of micro RNA-375 and epithelial-mesenchymal transition with the resistant phenotype [6]. We have also recently demonstrated the contribution of the alternative G-protein coupled estrogen receptor GPR-30 to the tamoxifen resistance phenotype [7,8].

Most cancer cells rely on aerobic glycolysis with subsequent lactate production and not further metabolism of pyruvate in the TCA cycle for their energy metabolism, the "Warburg effect" [9]. The increased flow of metabolites through gylcolysis is associated with an accumulation of side products such as the α-oxo-aldehyde methylglyoxal [10]. This molecule, together with the smaller glyoxal is responsible for the increased aldehyde stress observed in many cancer cells. An accumulation of α-oxo-aldehydes results in increased formation of "advanced glycation end products" (AGEs), which represent stable end products from the reaction of aldehydes with amino groups, the so-called Maillard reaction [11]. Here, an initially formed Schiff's base firstly undergoes the Amadori rearrangement to form early glycation products, which are then subject to further oxidations, rearrangements and eliminations. As a result, the AGEs represent a family of structurally diverse entities. They can be classified according to their physico-chemical properties, as for example, being fluorescent, such as arginine-pyrmidine (N^{δ}-(5-hydroxy-4,6-dimethylpyrimidine-2-yl)-L-ornithine: ArgPyr) or pentosidine and non fluorescent, such as the rather simple alkylation products N^{ε} -carboxy methyl lysine (CML) and N^{ε}-carboxyethyl lysine (CEL). Some of these AGEs form cross-links between amino acids in proteins. Frequently investigated examples for this group are pentosidine or glyoxal-lysine dimer (GOLD) [12]. Other authors have classified some AGEs according to their biological effects as toxic AGEs

(TAGEs) [13] which are also often referred to as glycotoxins [14]. AGE-modification of proteins influences their biological activity as enzymes [15] or signalling molecules [16] as well as their stability and degradation [17]. An increased cross linking of extracellular matrix proteins can also result in increased stiffness of organs such as the heart [18].

Additionally, the accumulation of reactive aldehydes and subsequent AGE-formation can influence gene expression or the activity of signal transduction molecules such as ion channels or growth factors [19,20]. Furthermore AGEs themselves can act as signalling molecules and increase oxidative stress and expression of proinflammatory cytokines through specific receptors such as RAGE (receptor for AGEs) [21,22]. As a consequence of these adverse effects, cancer cells depend on the expression of aldehyde defence enzymes, namely glyoxalase I (GLO1, EC 4.4.1.5) and –II (GLO2, EC 3.1.2.6) also called hydroxyacyl glutathione hydrolase (HAGH) [23] to avoid excessive aldehyde stress and fructosamine-3-kinase (FN3K, EC 2.7.1.171) [24] to prevent AGE-accumulation.

Overexpression of the alternative, membrane bound estrogen receptor GPR-30 (GPER) is one mechanism to overcome the growth inhibition by blocking the classical ER with tamoxifen [25]. GPR-30 is a typical G-protein coupled receptor that signals to phospholipase, which releases inositol triphosphate, and to adenylyl cyclase which produces cAMP [26]. cAMP is, besides other pathways, involved in the regulation of glycolysis and we therefore hypothesized that GPR-30 expressing cells might have increased glycolytic rates [27] which should result in altered formation of methylglyoxal and also aldehyde defence enzymes. In this study we therefore analysed whether tamoxifen resistant MCF-7 cells (TamR) indeed show an altered response towards exogenous methylglyoxal and glyoxal in terms of cell viability, MAP-kinase- and NF-κB activation and accumulation of AGEs. These data will potentially lead to the development of novel approaches to treat tamoxifen resistant breast cancer or to develop novel prognostic biomarkers for tamoxifen resistant ER-positive breast cancer.

Materials and Methods

Cells, antibodies and reagents

MCF-7 cells were obtained from the American tissue culture collection (ATCC, HTB-22) and tamoxifen resistant cells were developed by cultivating these cells continuously with 4-OH-tamoxifen (10 nM, Sigma-Aldrich, Munich, Germany) as previously described [8]. Tamoxifen resistant MCF-7 cells (TamR) were continuously grown in the presence of 4-OH tamoxifen (10 nM, Sigma-Aldrich, Munich, Germany). These MCF-7-Md and TamR-Md cells were cultivated in RPMI medium supplemented with FCS (10%, Biochrome, Berlin, Germany) and Penicillin/Streptomycin (Biochrome, Berlin, Germany).

MCF-7-Hd and TamR-Hd cells [6], were cultivated in phenol-red-free DMEM/F12 medium (Gibco, Darmstadt, Germany) supplemented with FCS (10%, Biochrome, Berlin, Germany) and Penicillin/Streptomycin (Biochrome, Berlin, Germany). MCF-7/S0.5 and MCF-7/TAM^{R}-1 cells (MCF-7-Dk and TamR-Dk) [28] were cultivated in the same medium but supplemented with 1% FCS and insulin (6 ng/mL, Sigma-Aldrich, Munich, Germany). TamR-Hd and TamR-Dk cells were continuously grown in the presence of 1 μM 4-OH tamoxifen. All cell lines were routinely screened for mycoplasma contamination.

Primary antibodies were obtained from Cell Signaling Technologies (via NEB, Frankfurt, Germany) for phosphorylated extracellular signal regulated kinase (pERK), pAKT, pp38MAPK pIκBα and IκBα or Sigma-Aldrich (β-actin). CML rabbit serum was raised against CML-modified key limpet hemocyanine [29,20]. The monoclonal antibody 6B directed against Arg-pyrimidine [30] was obtained from Biologo (Kronshagen, Germany). Secondary, peroxidase conjugated antibodies were from Dianova (Hamburg. Germany). Fluorescence labelled secondary antibodies (Dylight) were from Vector Laboratories (Biozol, Eching Germany). Methylglyoxal and glyoxal solutions were from Sigma-Aldrich (Munich, Germany). This methylglyoxal preparation was described to contain up to 9% formaladehyde [31].

Cell viability

Approximately 50,000 cells were seeded per well of a 24-well plate. After incubation for one day, medium was replaced by fresh medium containing either glyoxal, or methylglyoxal or solvent (NaCl 0.9%). After further 3 days, medium was replaced with 300 μl PBS containing Ca^{2+}- and Mg^{2+}-ions (Biochrome, Berlin, Germany) and resazurin (10 μg/mL) and incubated for further 30 to 120 minutes at 37°C. Then 100 μl resazurin solution was sampled and fluorescence recorded at wavelengths 525/580-640 nm (excitation/emission, fluorescence module "green") in a glomax microtitreplate reader (Promega, Mannheim, Germany). EC 50 values were determined from individual concentration-response by fitting experimental data to a sigmoidal equation using Origin 7.0 (Microcal Software, Northampton). EC-50 data are expressed as mean ± STD. The Student t test was used to analyze the differences between the groups. A value of $p<0.05$ was considered statistically significant.

Hypoxia treatment

For hypoxia treatment, cells were supplemented with fresh medium and transferred to a hypoxia incubator (Galaxy R, RS-Biotech) set to 0.1% oxygen for 24 h. After hypoxia treatment, cell cultures were taken from the incubator, growth medium was aspirated without any further delay and cell lysis was immediately carried out by addition of Trifast reagent (Peqlab, Erlangen, Germany) for subsequent RNA preparation.

RT-PCR

RNA was isolated according to the Trifast protocol (Peqlab, Erlangen, Germany). cDNA was synthesized from 500 ng RNA with oligo dT18 primer and Bioscript MMLV RNAse H^{-} reversed trancriptase (Bioline, Luckenwalde, Germany). Real-time PCR was performed using a light cycler 2.0 and Cybergreen master mix (Roche, Mannheim, Germany). Primers and annealing conditions are described in table 1. Identity of the PCR-products was controlled by melting curve analysis and agarose electrophoresis. Standard curves were prepared from isolated PCR products by serial dilution. Data were normalized towards α-tubulin-expression.

Cell stimulation experiments

Cells were grown to confluence in 24-well plates before being subjected to serum starvation for 48 h in RPMI medium without antibiotics and phenolred. Dicarbonyls, Il-1β (10 ng/ml), FCS (5%) or control solvent was added for the times indicated. Then medium was aspirated and cells immediately lysed with SDS-lysis buffer (TRIS/Cl 50 mM pH 6.8, SDS 2%, phosphatase- and protease inhibitor mix (Sigma-Aldrich, Munich, Germany)). MCF-7-Hd; MCF-7-Dk, TamR-Hd and TamR-Dk cells were treated in the same way but starvation was performed with DMEM/F12 medium without serum and phenol-red.

Table 1. Primers and amplification conditions used for RT-PCR.

Gene	accession	primer	product	annealing
GLO1	NM_006708.2	TGACCATTGTGCTCTTGGCT AGTACAAGCACGGTTGGCAT	395 bp	52°C
GLO2 (HAGH)	NM_005326.4 NM_001040427.1	CCAGCCCTGCTGGGAGTT CGACTCCAGCTTGACCAGTT	279 bp	52°C
FN3K	NM_022158.3	CATCCCGCAGGTGAATGAGT GAAGGAAGCCGGGTCGTAAA	256 bp	52°C
VEGF var. 1-9	NM_001025366.2	GGGCAGAATCATCACGAAGT TGGTGATGTTGGACTCCTCA	211 bp	58°C
GAPDH	NM_002046.4 NM_001256799.1	ATCATCCCTGCCTCTACTGG CCCTCCGACGCCTGCTTCAC	188 bp	57°C
α-tubulin	NM_032704.3 NM_006082.2	TCTTCAGGGCTTCTTGGTTT GGTGGTGAGGATGGAGTTGT	181 bp	56°C

doi:10.1371/journal.pone.0101473.t001

Western-blotting

Proteins were obtained by lysing the cells with SDS-based lysis buffer (see above). After denaturing SDS-polyacrylamide electrophoresis (12%), proteins were transferred to nitrocellulose by tank blotting for 90 min at 100 V in TRIS/glycine buffer supplemented with methanol (20%). After staining with poinceau red, detection of antigens was performed as described earlier [20]. Blocking reagent was either BSA (2%, for phosphospecific antibodies and CML), which was prescreened for possible interference with the CML detection due to high endogenous CML content, or Rotiblock (Carl Roth, Karlsruhe, Germany, for anti Arg-Pyr (6B)) in TBS containing NP-40 (0.2%) Chemiluminescence signal was detected with ECL-detection reagent (Millipore, Darmstadt, Germany) in a GeneGnome luminescence imager (Syngene, Cambridge, UK). Membranes were stripped with Restore Western blot stripping buffer (Thermo, Bonn, Germany) before reprobing. Signals were quantified by using the ImageJ software package [32] and normalized towards the β-actin signal from the same blot.

Immunofluorescence

Cells were grown on chamber slides (BD-Falcon, Heidelberg, Germany) until 50–80% confluence was reached. Dicarbonyls were added to the medium and after 24–48 h cells were fixed with methanol (10 min) and then acetone (5 min) at −20°C. Slides were blocked with 10% normal goat serum in PBS supplemented with 0.2% Triton X-100. Primary antibodies were incubated in PBS (BSA 1%) at 4°C overnight. After washing in PBS, fluorescent secondary antibody was used in the same buffer for 1 h at room temperature. After three further washes, slides were mounted with Vectashield mounding medium with DAPI (Vectorlabs, Biozol, Eching, Germany) and photographed using a Zeiss Axioplan 2 fluorescence microscope, equipped with a Plan Neofluar 40×/0.75 objective (Zeiss, Jena, Germany) and Zeiss filter sets 02 (G365 nm, FT 395 nm, LP 420 nm) 10 (BP 450–490 nm, FT 510 nm, BP 515–565 nm), and 15 (BP 546 nm, FT 580 nm, LP 590 nm). Photographs were taken with a JAI CV-M1 digital camera as part of the isis imaging system version 4.4.24 (MetaSystems, Altlussheim, Germany).

Cell fractionation

Logarithmically growing cells were harvested in ice-cold PBS by using a cell scraper and subcellular fractions were obtained with a subcellular fractionation kit (Calbiochem, Darmstadt, Germany) as previously described [33] and recommended by the manufacturer.

Caspase assays

Caspase activity assays were performed by using the caspase 3/7-glo luminescence assay system (Promega, Mannheim, Germany) and luminescence was read in a glomax multidetection system (Promega, Mannheim, Germany). Data were expressed relative to control treatments for each cell line.

Determination of free SH-groups (glutathione)

Free sulfhydryl (SH)-groups were determined by electron spin resonance using the biradical bis (2,2,5,5, tetramethyl 3-imidazolin 1-yloxy 4-yl) disulfide (100 µM) [34] as previously described [33]. Briefly, cells were grown to approximately 70% confluency in 6-well plates. Dicarbonyls were added for one hour at 1 mM concentration. Afterwards cells were washed twice with ice-cold phosphate buffered saline (PBS) and detached in 1 mL PBS by using a cell scraper. After centrifugation (1,000×g 15 min at 4°C) cells were resuspended in 100 µl sodium phosphate buffer (50 mM, pH 6.5) and stored at −80°C until further use. For measurement, cells were lysed with 0.1% Triton X-100, centrifuged (12,000×g, 5 min 4°C) and supernatants used for ESR and protein determination (BioRad DC protein assay, Munich, Germany). ESR spectra were recorded using a Miniscope 100 (Magnettech Berlin, Germany) using the following settings: center field: 3365 G, sweep width: 50 G, sweep time: 60 s, modulation amplitude: 0.1 G, power attenuation: 12 dB, receiver gain: 50. Each experiment was repeated 3 times and the data expressed as mean ± standard error of the mean (SEM). An external standard curve was prepared using glutathione. Additionally, dicarbonyls (1 mM) were added to the standards to assure that these substances do not interfere with the assay.

Determination of necrosis and apoptosis by flow cytometry

To discriminate between apoptosis and necrosis, combined staining with annexin V-fluoresceine conjugate and propidium iodide was performed by using the FITC Annexin V Apoptosis Detection Kit 1 (BD-Pharmingen, Heidelberg, Germany) according to the manufacturer's recommendations. Cells were seeded into 6-well plates to a density of about 50%. The next day, aldehydes were added and cells further incubated for 4–16 h as indicated. Afterwards, cells were detached with Trypsin/EDTA, stained and analyzed with a Fortessa cell analyzer (BD-Biosciences, Heidelberg, Germany).

Statistics

Statistical calculations were performed with the SPSS programme package vers. 21 (IBM, Ehningen, Germany). Details are indicated in the tables and figures.

Results

Toxicity of dicarbonyls was increased in tamoxifen resistant cells

To analyse whether the three independently generated tamoxifen resistant cell lines would differ in their respective aldehyde stress response we determined the viability/proliferation of these cells towards methylglyoxal and glyoxal. The applied resazurin assay determines the number of cells as well as their capability to reduce this dye. All three tamoxifen resistant cell lines were indeed more sensitive towards either glyoxal or both dicarbonyls. EC_{50} values were estimated to be 1.2±0.3 mM and 0.6±0.2 mM for glyoxal ($p = 8.2*10^{-4}$) and 0.6±0.1 and 0.4±0.1 mM for methylglyoxal ($p = 0.002$) for the MCF-7-Md and TamR-Md cell line, respectively (Figure 1). MCF-7-Hd and TamR-Hd cells tolerated higher concentrations of the aldehydes. No difference in toxicity was found for methylglyoxal (EC_{50} value: 0.8±0.3 mM for both cell lines, $p = 0.8$) but TamR-Hd cells were more sensitive to glyoxal (EC_{50} value 1.1±0.3 mM and 2.1±0.8 mM respectively ($p = 7.6*10^{-4}$). Regarding the Dk-cell lines, EC_{50} values for MCF-7-Dk and TamR-Dk were 1.0±0.4 mM and 0.8±0.1 mM for methylglyoxal ($p = 0.055$) and 1.9±0.2 mM and 1.1±0.5 mM for glyoxal ($p = 1.4*10^{-4}$), respectively. Hence, aldehyde toxicity was increased for both aldehydes in TamR-Dk cells.

Increased AGE-accumulation occurred under dicarbonyl stress

To analyze whether this increased toxicity correlated with increased AGE-accumulation, we then determined the amount of advanced glycation end products that accumulate in the MCF-7-Md and TamR-Md. The AGE $^{\varepsilon}$N-carboxymethyl lysine (CML) is a marker for glyoxal stress whereas methylglyoxal causes an increased formation of N^{δ}-(5-hydroxy-4,6-dimethylpyrimidine-2-yl)-L-ornithine (ArgPyr) and to a larger amount MG-H1 (5-Hydro-5-methylimidazolon). These AGEs were detected by specific antibodies and Western blotting.

The α-CML antiserum was raised against CML modified key limpet hemocyanine (KLH) and specifically detects glyoxal induced AGEs, especially CML [29]. The monoclonal antibody 6B was raised against methylglyoxal modified KLH [30]. It specifically detects ArgPyr but also cross reacts weakly with the MG-H1 antigen which is usually present in vast excess over ArgPyr. It is therefore expected that this antibody will detect both methylglyoxal induced AGEs (MG-AGEs). As we could not detect differences in AGE accumulation between MCF-7-Md and TamR-Md in total protein lysates (data not shown), we performed a subcellular fractionation. Only five differences in the pattern of AGE-modified proteins could be detected with this technique. In detail, a 60 kDa protein of the cytosolic fraction and three proteins of 19, 28 and 62 kDa of the nuclear fraction were more intensively stained for MG-AGEs in MCF-7-Md than in TamR-Md. For CML, two bands at 28 kDa of the nuclear as well the cytoskeletal fraction were more intense in the MCF-7-Md cells (indicated by arrows in figure 2). In a next set of experiments, we analysed whether increased exogenous dicarbonyl stress can increase the amount of AGE-modified proteins. This was indeed the case at concentrations above 1 mM (Figure 3A). This stress induced AGE-formation was specific for the dicarbonyl applied, as glyoxal produced CML and methylglyoxal resulted in MG-AGEs. This result could be further confirmed by immunofluorescence (Figure 3B). We were also able to detect AGE-modification of serum proteins of the growth medium at carbonyl concentrations higher than 1 mM in dot blots (data not shown). Both results showed that AGE-formation was not due to heating of the samples for denaturing electrophoresis.

Dicarbonyl toxicity was associated with nuclear condensation and caspase activation

To characterize the type of cell death further, we investigated nuclear morphology and caspase activity after dicarbonyl treatment in MCF-7-Md and TamR-Md cells. We observed a dose dependent condensation of the nuclei (Figure 4A). The activity of the executor caspases 3/7 was determined with a specific luminescent enzyme assay. As MCF-7 cells are deficient in caspase-3, [35] this assay will here only determine capase-7 activity. This caspase activity was highest at 6 h after adding the dicarbonyls to the cells (data not shown). At this time point, TamR-Md cells exhibited a higher increase in caspase activity than wt MCF-7-Md cells and glyoxal resulted in higher caspase activity than methylglyoxal. At very high (5 mM) concentration of the dicarbonyls, caspase activation was similar for both cell lines with 1.7-fold activation for glyoxal and 1.4-fold activation for methylglyoxal (Figure 4B). Annexin V and propidium iodide staining was performed to further discriminate necrotic cell death from apoptosis. Annexin V positive cells are considered to undergo apoptosis whereas necrotic cells are characterized by unlimited entry of propidium iodide and subsequent nuclear staining. This analysis was performed after 6 h of treatment when caspase activity was found to be highest with aldehydes at 2 mM concentrations. We then observed a small, but significant increase in the percentage of cells that were positive for annexin V, propidium iodide and both dyes (Figure 4C). Whereas glyoxal treated MCF-7-Md cells exhibited an increase in all three cell populations of about one third, methylglyoxal treatment resulted mainly in increased numbers of propidium iodide positive cells and even in a decrease in annexin V positive and propidium iodide negative cells (Figure 4C).

Dicarbonyl defence gene expression was comparable in MCF-7 and TamR cell lines

To explore the dicarbonyl defence capacity of the cells, the mRNA accumulation of glyoxalase-1 (GLO1) and -2 (GLO2) as well as of fructosamine 3-kinase (FN3K) was determined by quantitative RT-PCR under normal growth conditions and after hypoxia treatment and normalized to alpha-tubulin expression. We found no significant differences between the MCF-7-Md and TamR-Md cell lines (Table 2). Under hypoxic stress conditions we expected a higher glycolytic flow with higher methylglyoxal production, possibly resulting in increased defence gene expression. VEGF mRNA was used as a positive control. TamR-Md exhibited slightly reduced VEGF mRNA level already under normoxic conditions and VEGF mRNA accumulated only by a factor of 32 under hypoxia treatment, whereas MCF-7-Md showed a 92-fold increase. GAPDH was equally induced in these cell lines by a factor of approximately 7-fold. GLO-1 was also weakly induced by hypoxia in MCF-7-Md cells, whereas the TamR-Md- hypoxia response for these two genes was not significant. In case of FN3K, only TamR-Md cells exhibited increased mRNA accumulation under hypoxic conditions, mainly due to the lower basal expression under normoxia (Table 2). In further analysis, we analysed the mRNA accumulation of GLO1 and-2 as well as FN3K in MCF-7-Hd, and –Dk as well as in TamR-Hd and-Dk under control conditions. Again, no significant differences between these MCF-7 and TamR lines were observed (data not shown).

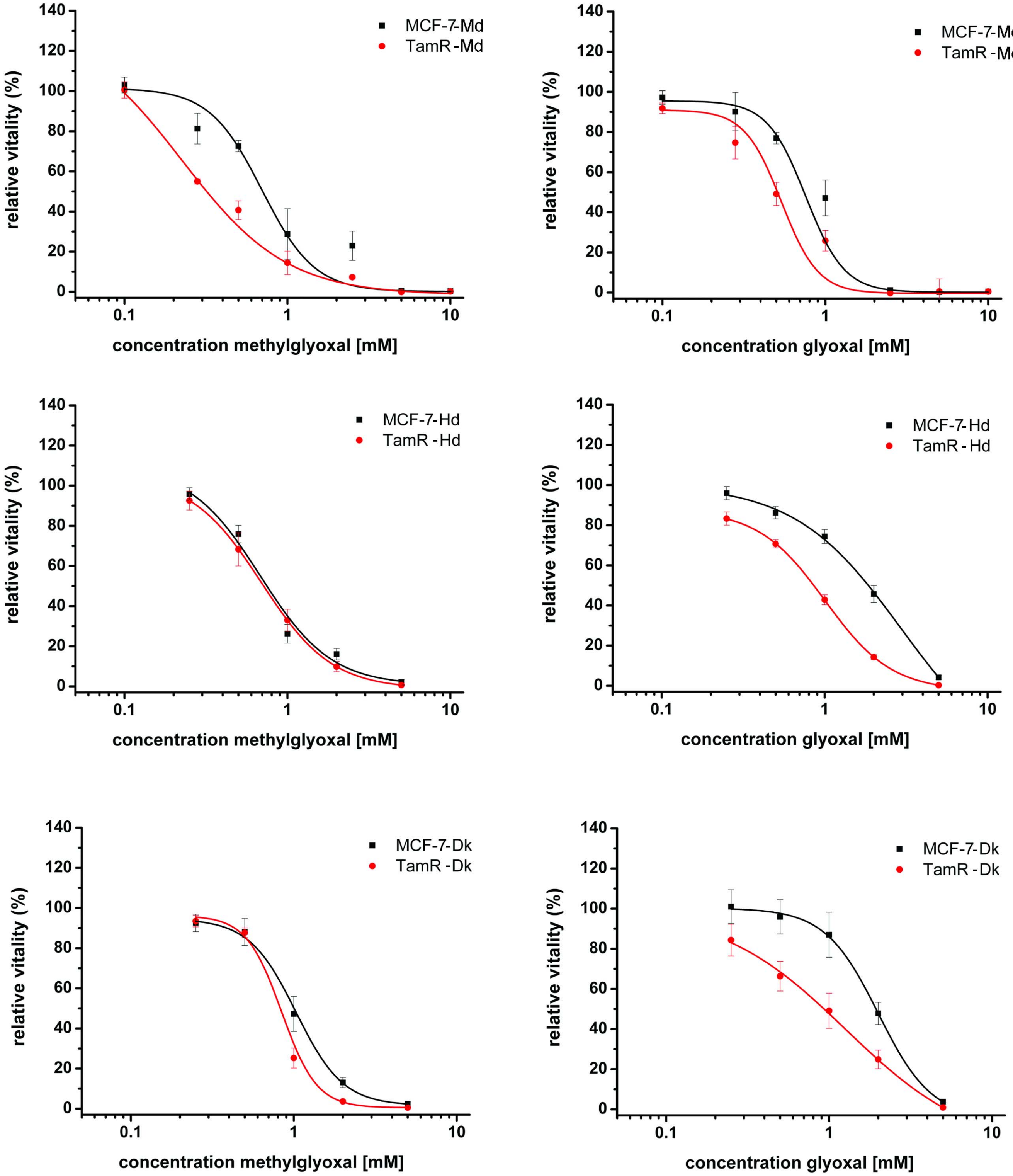

Figure 1. Vitality / proliferation of MCF-7 and TamR cells in response to treatment with methylglyoxal and glyoxal. Cells were cultivated with the indicated concentrations of the aldehydes for 3 days in full medium before proliferation and vitality was assessed by the resazurin assay. Signals form control cultures without added aldehydes were set to 1.0.
doi:10.1371/journal.pone.0101473.g001

Tamoxifen resistant cells contained less free SH-groups

Dicarbonyl defence by the glyoxalase system strongly relies on the presence of the cofactor glutathione (GSH) which is also needed for antioxidant defence. Other free sulfydryl groups do also act as antioxidants, and are an important depot for aldehydes as reversible binding of the aldehydes especially to cystein occurs

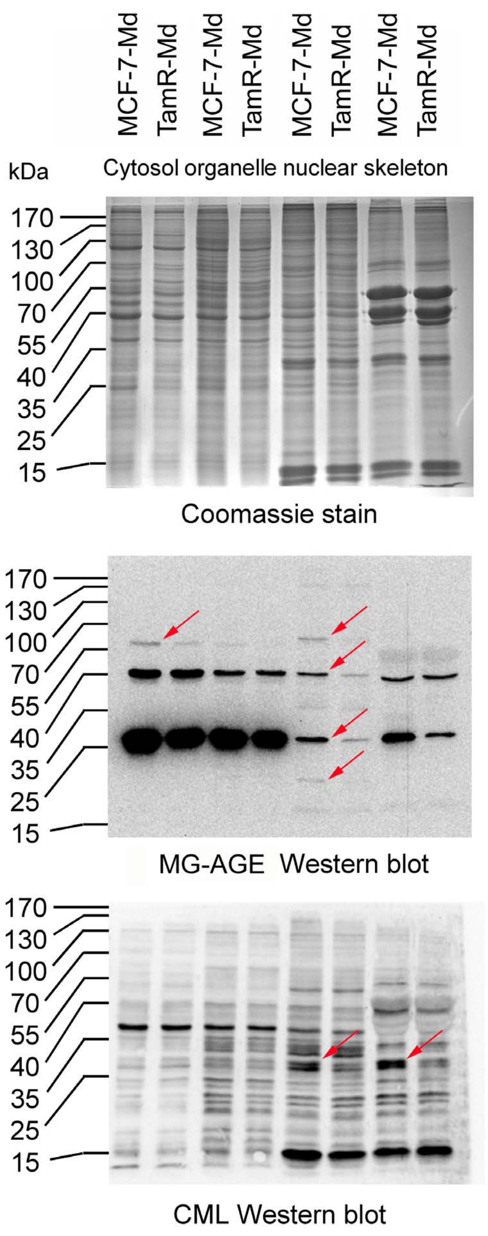

Figure 2. AGE accumulation in MCF-7 and TamR cells. MCF-7-Md and TamR-Md cells were grown under standard conditions and harvested in the logarithmic phase. Cells were fractionated by a detergent based protocol and AGEs detected by Western blotting. Based on protein determination and Coomassie staining (A), equal amounts of protein were loaded for both cell lines and the AGEs MG-AGE (B) and CML (C) detected by Western analysis. Differences in the pattern of AGE-modified proteins are indicated by arrows.
doi:10.1371/journal.pone.0101473.g002

[36]. Therefore, we determined the amount of free sulfhydryl groups by ESR spectroscopy as such free SH-groups are mainly provided by glutathione. Nevertheless other antioxidants such as thioredoxins will also contribute to these data. TamR-Md cells contained only about 60% of the SH-groups determined in MCF-7-Md cells and this was further decreased after 1 h dicarbonyl treatment (Table 3).

Dicarbonyl stress resulted in MAP-kinase and NF-κB activation

To analyze early signalling events that were involved in the response towards aldehydes, we determined the phosphorylation of the MAP-kinases p38 and p42/44 (ERK1/2) as well as protein kinase B (AKT) by Western blotting (Figure 5). The MCF-7-Md and TamR-Md cell lines exhibited a clearly different dose response curve and responded significantly stronger to methylglyoxal than glyoxal treatment. TamR-Md cells were particularly more sensitive towards methylglyoxal than the parental MCF-7-Md cell line, especially in case of AKT and p38-MAPK-phosphorylation. Interestingly, the 5 mM concentration of methylglyoxal, strongly reduced kinase phosphorylation in TamR-Md cells only (Figure 5). We then determined the phosphorylation of the NF-κB subunit IκBα by Western blotting as an indication for NF-κB activation. Only TamR-Md cells responded with IκBα phosphorylation to exogenous methylglyoxal but not glyoxal stress (Figure 6). TamR-Md cells also contained about half IκBα than the MCF-7-Md parental cell line (MCF-7: 1.0±0.1; TamR: 0.52±0.04 $p<0.001$; normalized to β-actin and MCF-7) which argues for higher basal activity of NF-κB in these cells.

MCF-7-Hd and TamR-Hd cells challenged with the aldehydes responded also with increased IκBα-, p38-MAPK- as well as ERK1/2- and AKT-phosphorylation (Figure 7A). The responses towards glyoxal were rather weak compared to the challenge with methylglyoxal. In fact, MCF-7-Hd cells did not show significant changes in the phosphorylation events in respone to glyoxal at the time point analysed. Especially, IκBα-phosphorylation could only be achieved by stimulation with methylglyoxal and TamR-Hd cells showed this phosphorylation already at concentrations as low as 0.5 mM whereas for MCF-7-Hd cells, an increased phosphorylation was only seen with 2 mM methylglyoxal.

MCF-7-Dk and TamR-Dk cells showed less pronounced phosphorylation events (Figure 7B). Neither glyoxal nor methylglyoxal treatment resulted in phosphorylation of IκBα, but the basal level of phosphorylated IκBα was higher in TamR-Dk cells compared to MCF-7-DK cells. pAKT was slightly increased in reponse to glyoxal and more significantly induced by methylglyoxal and this was more pronounced in TamR-Dk cells. p38-MAPK phosphorylation was slightly increased in response to glyoxal in TamR-Dk cells and not in MCF-7-Dk cells. Methylglyoxal resulted in increased p38-MAPK phosphorylation in both cell lines, but TamR-Dk cells responded at lower concentrations than the MCF-7-Dk cell line. For p42/44 (ERK1/2) a high degree of basal phosphorylation was observed in the TamR-Dk cell line in agreement with previously published data [28]. This was also evident in case of the MCF-7-Hd and TamR-Hd cell lines. Phosphorylation of ERK was only increased in response to methylglyoxal and this response was again stronger in the TamR-Dk cell line.

Discussion

Most cancer cells predominantly rely on aerobic glycolysis for energy production. As a side product of this increased glycolytic flow, methylglyoxal is produced in significant amounts and many cancer cells have increased expression of aldehyde defence genes such as glyoxalase-1 [10]. There is also evidence for persistent oxidative stress in cancer cells [37], which results in increased glyoxal levels from gycoxidation. It has therefore been suggested, that glyoxalase is a promising target for anticancer drugs [38].

Tamoxifen resistance poses an important problem in the treatment of hormone sensitive breast cancer and new strategies against these resistant cancer cells are urgently needed [39,40]. To

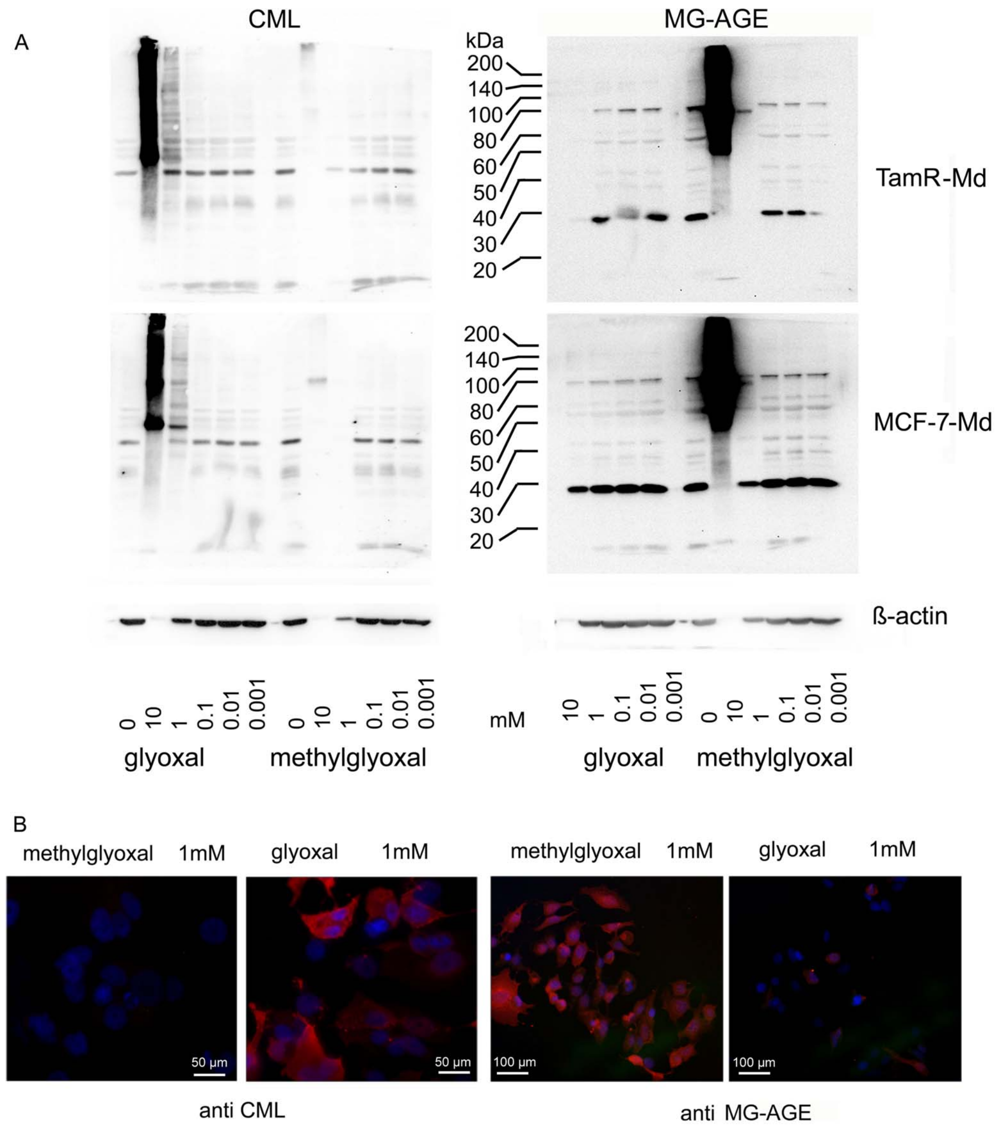

Figure 3. AGE accumulation under exogenous aldehyde stress. A: AGE accumulation (CML (left) and MG-AGE (right)) in MCF-7-Md (lower panel) and TamR-Md cells (upper panel) after cultivation for three days with different concentrations of methylglyoxal and glyoxal as shown by Western blotting. Strongly enhanced AGE accumulation became visible when cell number was significantly reduced due to toxic effects, as represented by the decreasing β-actin signal. Glyoxal resulted in accumulation of CML whereas methylglyoxal treatment caused MG-AGE modification. Cells were treated as described for the determination of vitality/proliferation. Proteins were extracted per well and not corrected for protein amount. Therefore, the blots represent adherent cells only. B) AGE accumulation shown by immunofluorescence. Composite images of dual exposures are shown. MCF-7-Md cells were stained for CML and MG-AGE (red) by specific antibodies as indicated and nuclear staining was achieved with DAPI (blue) of cells treated for 24 h with 1 mM methylglyoxal or glyoxal.
doi:10.1371/journal.pone.0101473.g003

analyze tamoxifen resistance *in vitro*, tamoxifen tolerant cell lines derived from the commonly used ER-positive breast cancer cell line MCF-7 [41] are a well established cellular model. In this study, we have primarily analyzed the TamR-Md cell line in which the alternative estrogen receptor GPR-30 contributes to the resistant phenotype [7,8]. GPR-30 signals through G-proteins to increase cAMP, Ca^{2+} and inositol-3-phosphate as second messengers. As cyclic AMP is well known to regulate glycolytic enzymes and to interact with other pathways, we hypothesized that the response to glycolytic side products such as methylglyoxal or the related C_2-moiety glyoxal might be more generally changed as a result of altered estrogen signalling in acquired tamoxifen resistance.

As a first indication that the TamR cells might respond differentially to aldehyde stress, we observed a moderate, approximately two-fold increase in sensitivity in terms of cell viability towards both α–oxo-aldehydes tested (Figure 1). An increased formation of dicarbonyls can result in increased accumulation of AGEs, in case that the dicarbonyl defence is not sufficient. However, we found only a few differences in AGE-modified intracellular proteins in the two cell lines under normal growth conditions (Figure 2). Nevertheless, these differences are too small to be a result of a broadly increased AGE-stress and might reflect minor differences in the two cell types such as a higher expression of the AGE-target proteins. We have already made similar observations with the osteosarcoma cell line 143b. These cells were also capable of keeping their AGE load virtually

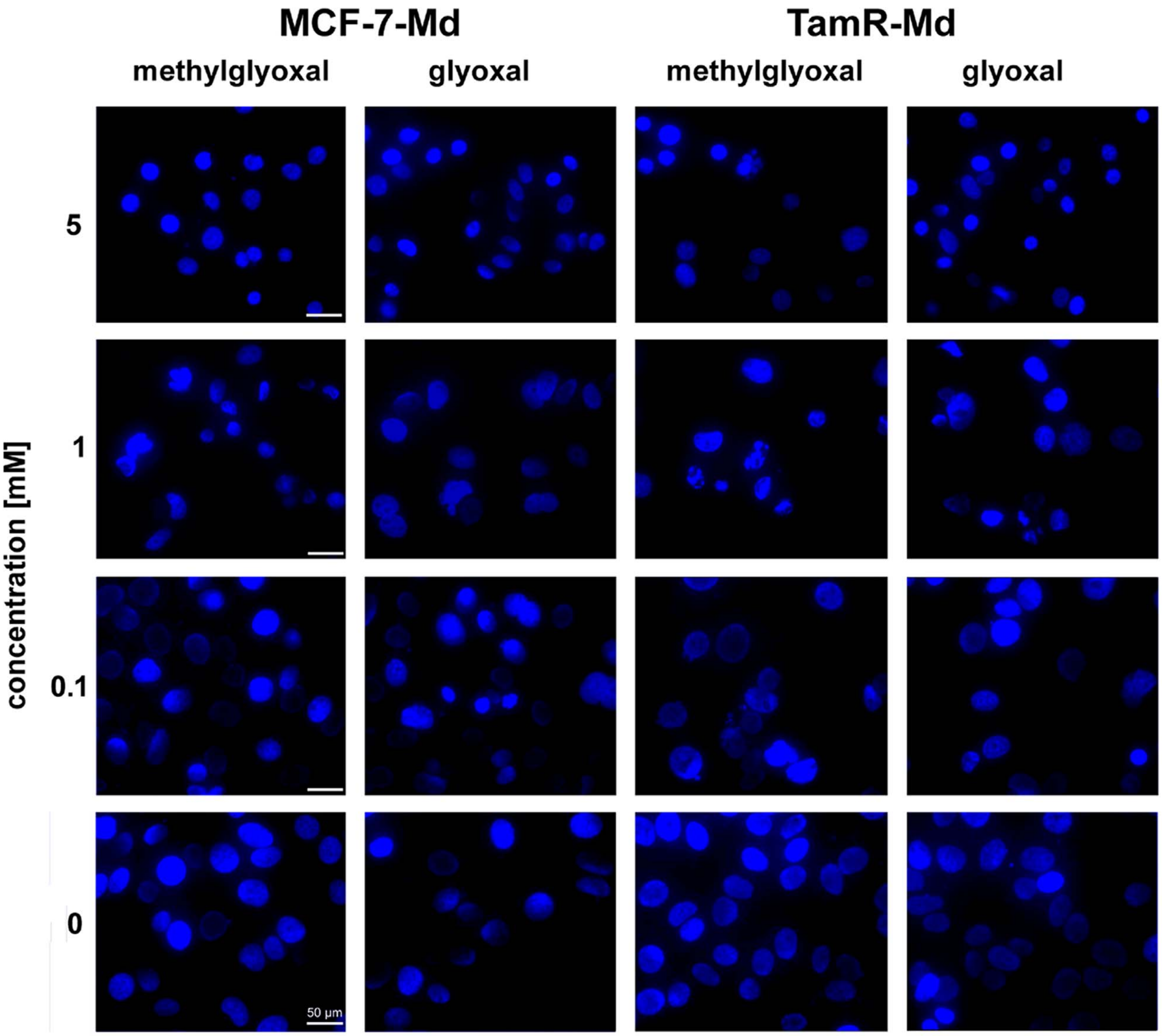
MCF-7-Md
TamR-Md
methylglyoxal
glyoxal
methylglyoxal
glyoxal
concentration [mM]
5
1
0.1
0
50 µm

4A

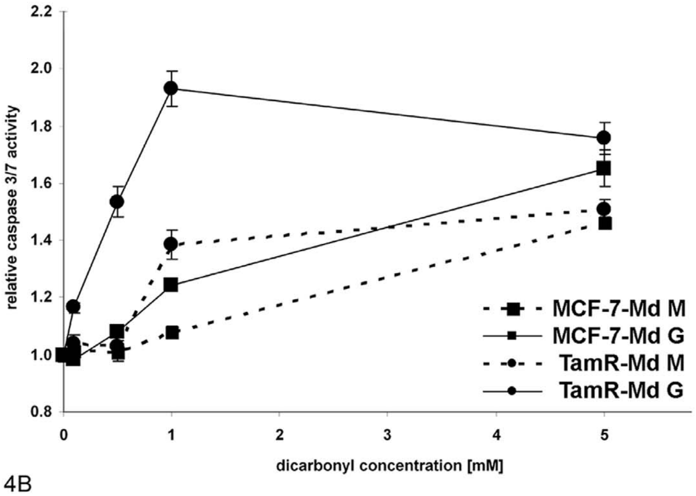
relative caspase 3/7 activity
dicarbonyl concentration [mM]
MCF-7-Md M
MCF-7-Md G
TamR-Md M
TamR-Md G

4B

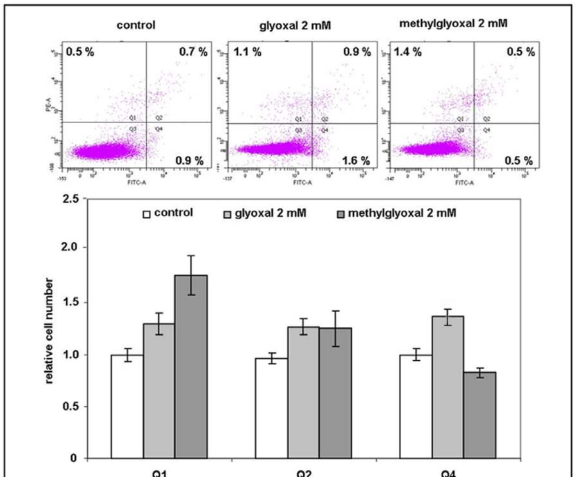
control
glyoxal 2 mM
methylglyoxal 2 mM
0.5 %
0.7 %
0.9 %
1.1 %
0.9 %
1.6 %
1.4 %
0.5 %
0.5 %
relative cell number
Q1
Q2
Q4

4C

Figure 4. Dicarbonyl treatment caused nuclear condensation and caspase 3/7 activation but resulted mainly in necrotic cell death. 4A: Assessment of nuclear morphology. MCF-7-Md and TamR-Md cells were incubated for 48 h with dicarbonyls at the indicated concentrations and nuclei visualised by DNA staining with DAPI and fluorescence microscopy. **4B: Determination of caspase activity.** MCF-7-Md and TamR-Md cells were incubated with the dicarbonyls for 6 h at the indicated concentrations. Executer caspases 3/7 were determined by a specific luminescent enzyme activity assay. Activity in control treatments of the respective cell line was set to 1. 4C: **Determination of necrosis and apoptosis by flow cytometry.** MCF-7-Md cells were treated for 6 h with 2 mM aldehydes before annexin V and propidium iodide double staining and flow cytometric analysis was performed. Representative results of the flow cytometry are shown on top and quantitative analysis of data from 7 experiments performed in triplicate is shown below. Percentage of cells in each quadrant in the control treatments was set to one. Annexin staining is shown on the y-axis and propidium iodide staining on the x-axis.
doi:10.1371/journal.pone.0101473.g004

constant although glycolytic and mitochondrial activity and, thus, aldehyde and oxidative stress were severely altered in mitochondria deficient 143b^{rho0} cells [33].

Only under severe exogenous dicarbonyl stress, a significant increase of AGE-formation in MCF-7 and tamoxifen resistant cells could be observed. Under these concentrations the cells showed reduced viability, demonstrating that the aldehyde defence systems were unable to cope with these intense noxes.

However, we found no striking differences in the expression of defence enzymes against dicarbonyls (glyoxalases) and early glycation products (FN3K) [24] even under hypoxic stress in the two cell lines (Table 2). But when analysing free sulfhydryl groups representing mainly glutathione, we found a significantly lower content in the tamoxifen resistant cell line and this was further reduced by exogenous dicarbonyl stress (Table 3) This reduced –SH content is important for the aldehyde stress response as on one hand glyoxalases need GSH as a cofactor for detoxification and on the other hand dicarbonyl stress is known to cause the production of reactive oxygen species by NADPH oxidases. This oxidative stress can then induce apoptosis [42–44]. In accordance with the literature, we have preliminary data by applying the redox sensitive dye 2′,7′-dichlorodihydrofluorescein diacetate (DCFDA) that show that dicarbonyls indeed evoke oxidative stress in MCF-7-Md and TamR-Md cells. Also, co-incubation with the antioxidant N-acetyl cystein (NAC) and the NADPH-oxidase inhibitor diphenyleniodonium (DPI) reduced the formation of reactive oxygen species and NAC also increased the viability of the cells (N. Nass, unpublished data).

Other known signalling pathways associated with dicarbonyl stress involve p38-MAPK [44], p42/44-MAPK [45], apoptosis [46], p21 activated kinase [45,46] and receptor kinases [47]. But also the intracellular kinase AKT1 can respond directly to methylglyoxal modification by increased phosphorylation [48,49]. We therefore analysed some aspects of signalling and apoptosis in the wildtype MCF-7 and derived TamR cell lines. Our results for MCF-7 and TamR cells are in accordance with these earlier reports, as we observed increased MAPK phosphorylation (Figure 5), caspase 7 activity (Figure 4B) and also activation of the ROS-sensitive NF-κB transcription factor, especially under methylglyoxal stress (Figure 6). All these events were significantly more pronounced in the TamR cell lines. The observed activation of caspase 7 and nuclear condensation were consistent with apoptotic cell death, although MCF-7 cells have been reported to be deficient in caspase 3 and are therefore not able to display all features typical for apoptosis, especially shrinking and blebbing [35]. However, as demonstrated by flow cytometric analysis of annexin V, as a marker for apoptosis and propidium iodide permeability, as a marker for necrosis, we mainly observed cells undergoing necrosis. Only in case of glyoxal, an increase in annexin V positive cells was also observed (Figure 4C). This correlated with the higher caspase activation in glyoxal than in methylglyoxal treated cells. In contrast to other cells, that undergo reactive oxygen/p38-MAPK induced apoptosis in response to aldehydes, we conclude that MCF-7, as being partially apoptosis deficient, exhibited mainly necrotic cell death in response to aldehyde stress.

As we have demonstrated, a decreased amount of free sulfhydryl groups in the TamR-Md line, we propose that TamR cells were more vulnerable to dicarbonyl stress because these limited amounts of glutathione may be insufficient for the glyoxalase system as well as antioxidant defence. This is consistent with increased activation of p38 MAPK and NF-κB, both representing ROS sensitive signalling molecules. Nevertheless, the reason for the decrease in free sulfhydryl groups is unknown and requires further investigations.

We would also like to point out that the two dicarbonyls clearly evoked different responses in the cells. For example, we obtained no evidence for IκBα phosphorylation in response to glyoxal which we observed in response to methylglyoxal. Additionally, caspase 3 activation was more pronounced in response to glyoxal.

Table 2. Relative mRNA accumulation of genes involved in dicarbonyl defence and hypoxia.

Genes	MCF-7 normoxia	TamR normoxia	MCF-7 hypoxia	TamR hypoxia
GLO1	1.0+/−0.14	1.59+/−0.30	2.03+/−0.36 $	1.11+/−0.24
GLO2	1.0+/−0.15	0.97+/−0.16	1.50+/−0.19	0.79+/−0.28
FN3K	1.0+/−0.19	0.55+/−0.10	1.16+/−0.37	1.36+/−0.28 $
VEGF	1.0+/−0.08	0.75+/−0.18	91.9+/−30.1 *	24.0+/−5.9 *§
GAPDH	1.0+/−0.07	0.96+/−0.15	7.44+/−1.13 *	6.68+/−1.27 *

Cells were cultivated for 24 h under normoxic or hypoxic conditions and mRNA accumulation determined by qRT-PCR as described in material and methods. mRNA accumulation was normalized towards α-tubulin mRNA content and MCF-7 under normoxic conditions was set to 1. Average and standard error (SEM) are given. Experiment was performed in triplicate with each data point determined with six repetitions each.
Statistics (one way ANOVA, Tamhane T2, post hoc analysis):
*: $p<0.05$ versus same cell line control.
$: $p<0.1$ versus same cell line control.
§: $p=0.1$ versus other cell line, same treatment.
doi:10.1371/journal.pone.0101473.t002

Table 3. Free SH-groups as a measure for glutathione in MCF-7 and TamR cells as determined by electron spin resonance spectroscopy.

Cells / treatment	Free SH groups pmol/μg protein
MCF-7 control	23.9±1.8
MCF-7 glyoxal	18.8±2.4
MCF-7 methylglyoxal	18.1±2.4
TamR control	13.6±2.2 *
TamR glyoxal	4.6±2.5 a
TamR methylglyoxal	6.3±1.7 b

Cells were grown in RPMI / FCS medium to about 70% confluence before aldehydes were added at 1 mM concentration for 1 h. Experiment was performed three times with duplicates for each treatment. Average and standard error are given. Statistical significance was determined by one way ANOVA after normal distribution was proven by Kolmogorov-Smirnov test.
*: significant to MCF-7 control; a: p = 0.06 to TamR control; b: p = 0.078 to TamR control.
doi:10.1371/journal.pone.0101473.t003

Although such differences in the responses of cells towards the dicarbonyls glyoxal and methylglyoxal have been discussed before [50], the reasons remain still unclear. Glyoxal and methylglyoxal differ only in one methyl group and this results in the formation of hydrates with differing reactivity [51]. The two molecules have also slightly different lipophilicity (logK Oc/w = −1.5 and −1.66, respectively (HSDB database http://toxnet.nlm.nih.gov/cgi-bin/sis/htmlgen?HSDB) and might therefore enter the cells with different efficiency. It is therefore plausible that these two molecules have also different target molecules, which should be the focus of further research.

A possible problem in interpreting the differential responses of the cells towards the aldehydes is the known contamination of commercial methylglyoxal with formaldehyde [31]. This substance is significantly more toxic than methylglyoxal and can therefore contribute to the observed high toxicity of methylglyoxal if

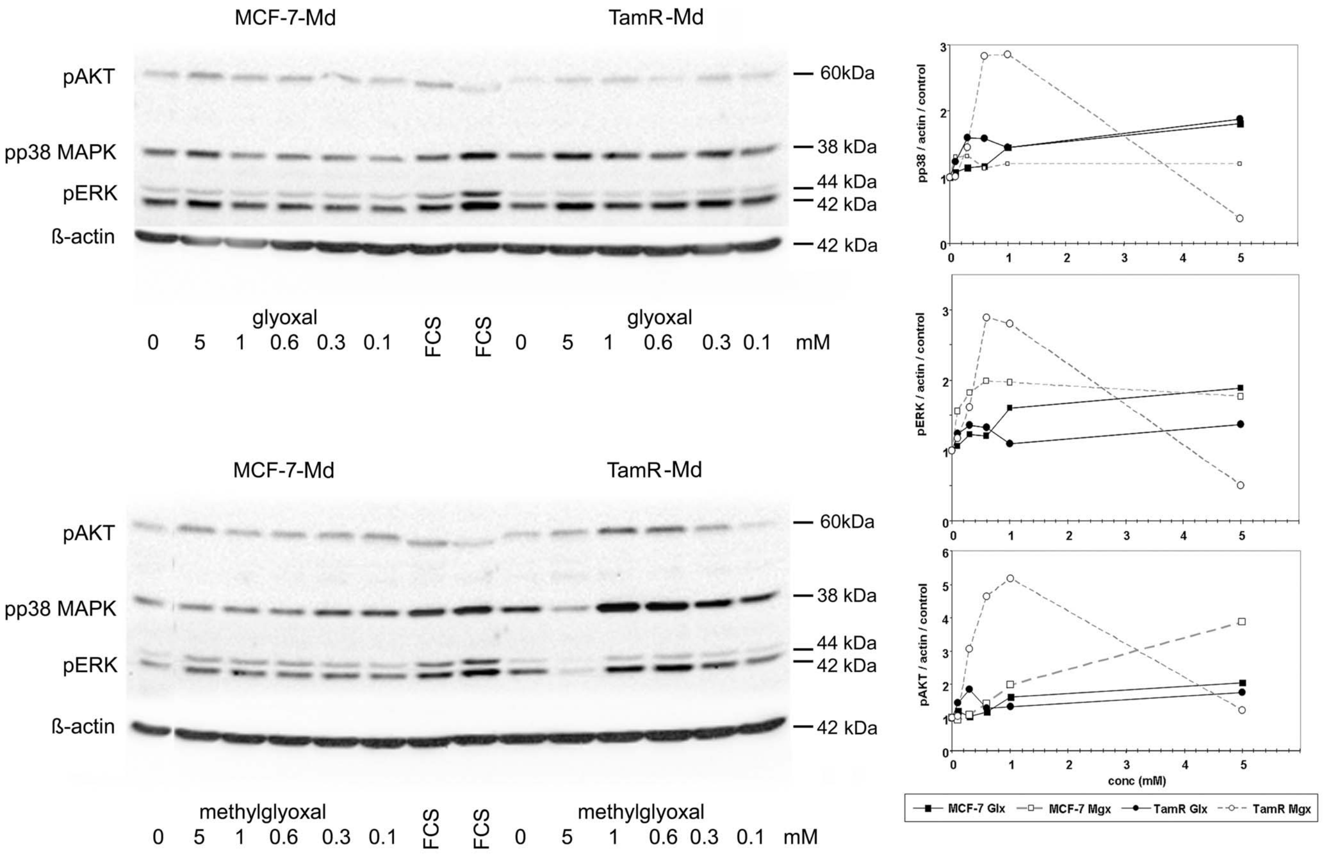

Figure 5. Kinase phosphorylation in MCF-7-Md and TamR-Md cells in response to glyoxal and methylglyoxal. Confluent and serum starved cells were incubated with different concentrations of dicarbonyls for 10 minutes before proteins were extracted and subjected to Western blot analysis. Experiments were repeated three times in duplicate. A representative Western blot and the quantification of the signals of all experiments are shown. Band intensity was expressed relative to the β-actin signal and control treatments were set to 1 for each cell-line.
doi:10.1371/journal.pone.0101473.g005

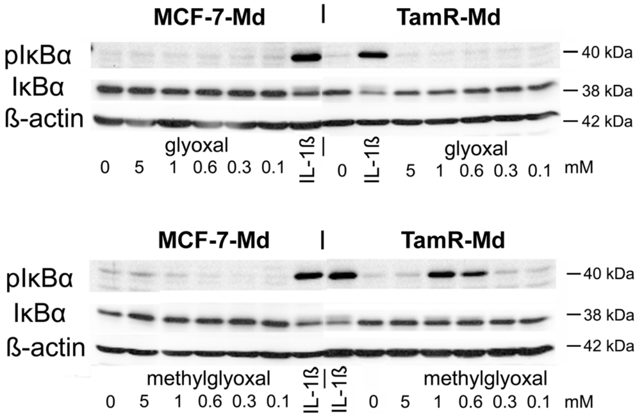

Figure 6. Phosphorylation of IκBα in response dicarbonyl stress. Cells were incubated with different concentrations of dicarbonyls and with Il-1β (10 ng/mL) as positive control treatment for 10 minutes. IκBα phosphorylation was detected by Western blotting. Experiment has been performed three times with duplicates for each treatment, one representative blot is shown.
doi:10.1371/journal.pone.0101473.g006

compared to glyoxal. However, when we challenged the cells with formaldehyde alone, we could not observe increased kinase signalling (data not shown), thus it is unlikely that the formaldehyde contamination was responsible for the different cellular responses towards methylglyoxal and glyoxal.

When we extended our study to independently generated tamoxifen resistant cell lines several similarities but also a clear differences were identified. The cell lines used here came from different laboratories and the tamoxifen resistant cell lines have been selected under different conditions. The TamR-Hd cells were generated very similarly to the TamR-Md line except that a richer growth medium and a 100-fold higher 4-OH-tamoxifen concentration have been used. The TamR-Dk cells were derived from a MCF-7 line that has been adapted to grow with reduced serum supplementation. These cells were also grown in a richer medium and the tamoxifen resistant cell line was established form a colony of cells surviving long term treatment with 1 μM tamoxifen [28,49].

Several possible mechanisms for tamoxifen resistance have been described, and the cell lines have each been studied with a different focus. TamR-Hd cells have been analysed under the aspect of micro RNA expression and an accompanying endothelial mesenchymal transition associated with tamoxifen resistance [6]. TamR-Dk cells were analysed with the focus on human epidermal growth factor receptors (HERs), ERK signalling and estrogen

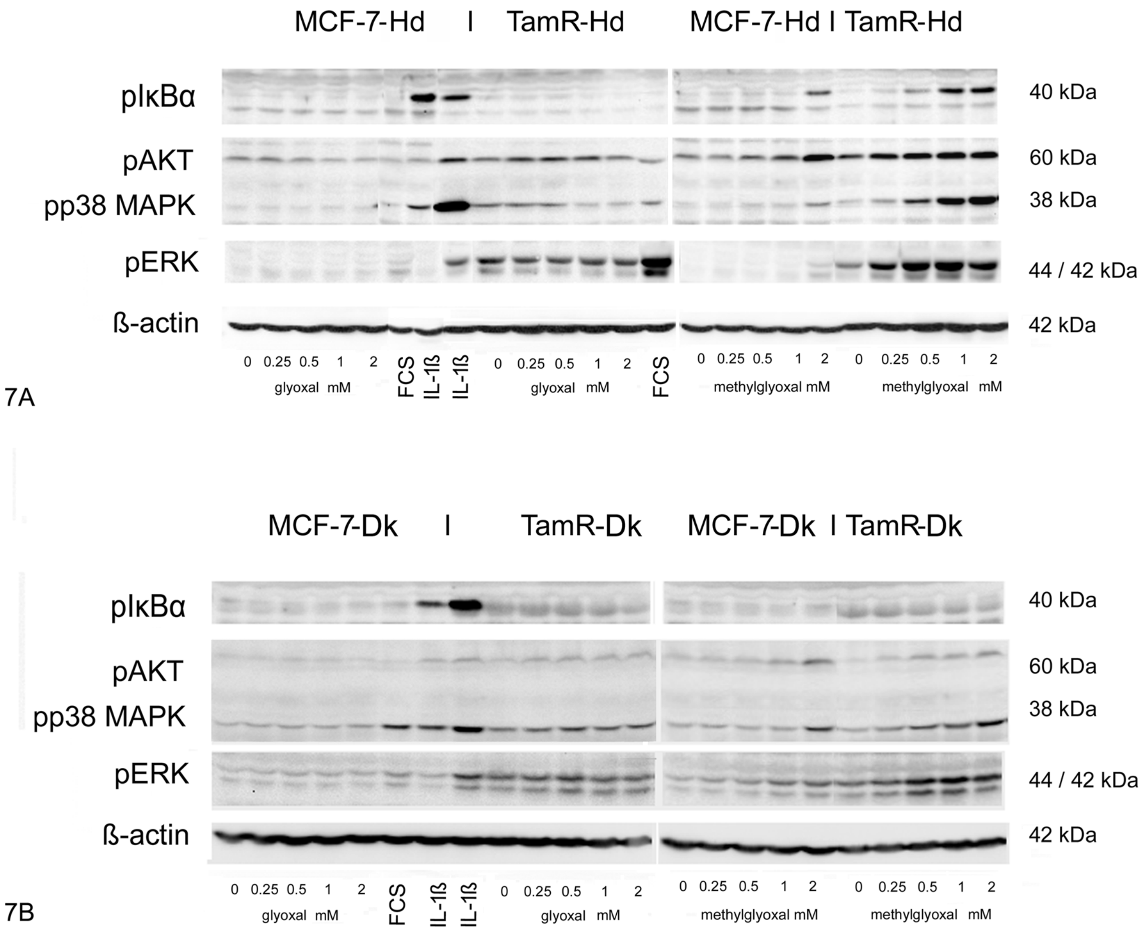

Figure 7. Phosphorylation of kinases in MCF-7-Hd and –Dk and TamR-Hd and -Dk cell lines in response to dicarbonyls. 7A: MCF-7-Hd and TamR-Hd cell lines 7B: MCF-7-Dk and TamR-HDk cell lines. Cells were treated as described for figure 5 and 6. Experiments were performed at least three times in duplicate and the result of one representative experiment is shown.
doi:10.1371/journal.pone.0101473.g007

receptor α [28,52]. For TamR-Md cells, we have intensively studied the contribution of GPR-30 to tamoxifen resistance [8]. Nevertheless, all TamR cell lines tested here exhibited an altered sensitivity towards the α-oxoaldehydes glyoxal and methylglyoxal if compared to the corresponding parental wt MCF-7 cell lines. It is tempting to propose that there is a common mechanism underlying this result. Our data strongly suggest that tamoxifen resistant cells are less resistant to oxidative stress, which can be caused by increased endogenous production of reactive oxygen species or a reduced expression of antioxidants defence systems. Clearly, further research is needed to clarify this point.

The relevance of these cell culture based results for tamoxifen resistance occurring *in vivo*, remains also to be elucidated. *In vivo*, the physiological concentrations of the dicarbonyls are usually in the micromolar range, nevertheless this can have severe implications for disease development and progression as shown i.e. for diabetes [19]. In contrast, the toxic exogenous concentration for these aldehydes in cell culture is usually in the millimolar range, depending on the cell type studied. However, in such experiments dicarbonyls are usually applied extracellularly and due to their fast, but reversible reaction with amines to form Schiff's bases, the amount of free aldehyde is much lower. Additionally, the intracellular concentrations of endogenous free aldehydes are unknown and will be highest in areas where glycolytic intermediates are localized.

Nevertheless, other groups have demonstrated that AGEs accumulate in mamma carcinoma [53] and that glyoxalase expression [54] and polymorphisms are relevant for breast cancer [55]. Consequently, targeting glyoxalase has been proposed as cancer therapy [10,56] and even oral consumption of methylglyoxal has been investigated in mice to treat cancer [57]. If the results presented here, are indeed representative for at least a subset of ER-positive, tamoxifen resistant mamma carcinomas, targeting glyoxalase might be a promising approach for the treatment of tamoxifen resistant ER-positive breast cancer. Furthermore, the carbonyl defence enzymes and AGE-modifications should be evaluated as putative prognostic or even predictive biomarkers.

Acknowledgments

The authors wish to thank Kerstin Werner and Martina Stoklasek for excellent technical assistance.

Author Contributions

Conceived and designed the experiments: NN FH AS AR TK. Performed the experiments: NN H-JB RH FH. Analyzed the data: NN H-JB RH SK SS TK. Contributed reagents/materials/analysis tools: NN H-JB RH AS FH AW SW AEL AR TK. Wrote the paper: NN RH SW AEL TK.

References

1. Clemons M, Danson S, Howell A (2002) Tamoxifen ("Nolvadex"): a review. Cancer Treat Rev 28: 165–180.
2. Kurebayashi J (2005) Resistance to endocrine therapy in breast cancer. Cancer Chemother Pharmacol 56 Suppl 1: 39–46.
3. Knowlden JM, Hutcheson IR, Jones HE, Madden T, Gee JMW, et al. (2003) Elevated levels of epidermal growth factor receptor/c-erbB2 heterodimers mediate an autocrine growth regulatory pathway in tamoxifen-resistant MCF-7 cells. Endocrinology 144: 1032–1044.
4. Vienonen A, Miettinen S, Manninen T, Altucci L, Wilhelm E, et al. (2003) Regulation of nuclear receptor and cofactor expression in breast cancer cell lines. Eur J Endocrinol 148: 469–479.
5. Zhou Y, Yau C, Gray JW, Chew K, Dairkee SH, et al. (2007) Enhanced NF kappa B and AP-1 transcriptional activity associated with antiestrogen resistant breast cancer. BMC Cancer 7: 59.
6. Ward A, Balwierz A, Zhang JD, Küblbeck M, Pawitan Y, et al. (2013) Re-expression of microRNA-375 reverses both tamoxifen resistance and accompanying EMT-like properties in breast cancer. Oncogene. 32(9):1173–82.
7. Ignatov A, Ignatov T, Weissenborn C, Eggemann H, Bischoff J, et al. (2011) G-protein-coupled estrogen receptor GPR30 and tamoxifen resistance in breast cancer. Breast Cancer Res Treat 128: 457–466.
8. Ignatov A, Ignatov T, Roessner A, Costa SD, Kalinski T (2010) Role of GPR30 in the mechanisms of tamoxifen resistance in breast cancer MCF-7 cells. Breast Cancer Res Treat 123: 87–96.
9. Upadhyay M, Samal J, Kandpal M, Singh OV, Vivekanandan P (2012) The Warburg effect: Insights from the past decade. Pharmacol Ther. 137(3):318–30
10. Thornalley PJ, Rabbani N (2011) Glyoxalase in tumourigenesis and multidrug resistance. Semin Cell Dev Biol 22: 318–325.
11. Nass N, Bartling B, Navarrete Santos A, Scheubel RJ, Börgermann J, et al. (2007) Advanced glycation end products, diabetes and ageing. Z Gerontol Geriatr 40: 349–356.
12. Glomb MA, Pfahler C (2001) Amides are novel protein modifications formed by physiological sugars. J Biol Chem 276: 41638–41647.
13. Sato T, Iwaki M, Shimogaito N, Wu X, Yamagishi S-I, et al. (2006) TAGE (toxic AGEs) theory in diabetic complications. Curr Mol Med 6: 351–358.
14. Cai W, He JC, Zhu L, Chen X, Zheng F, et al. (2008) Oral glycotoxins determine the effects of calorie restriction on oxidant stress, age-related diseases, and lifespan. Am J Pathol 173: 327–336.
15. Ahmed N, Dobler D, Dean M, Thornalley PJ (2005) Peptide mapping identifies hotspot site of modification in human serum albumin by methylglyoxal involved in ligand binding and esterase activity. J Biol Chem 280: 5724–5732.
16. Hunter SJ, Boyd AC, O'Harte FPM, McKillop AM, Wiggam MI, et al. (2003) Demonstration of glycated insulin in human diabetic plasma and decreased biological activity assessed by euglycemic-hyperinsulinemic clamp technique in humans. Diabetes 52: 492–498.
17. Bansode SB, Chougale AD, Joshi RS, Giri AP, Bodhankar SL, et al. (2013) Proteomic analysis of protease resistant proteins in the diabetic rat kidney. Mol Cell Proteomics 12: 228–236.
18. Van Heerebeek L, Hamdani N, Handoko ML, Falcao-Pires I, Musters RJ, et al. (2008) Diastolic stiffness of the failing diabetic heart: importance of fibrosis, advanced glycation end products, and myocyte resting tension. Circulation 117: 43–51.
19. Bierhaus A, Fleming T, Stoyanov S, Leffler A, Babes A, et al. (2012) Methylglyoxal modification of Nav1.8 facilitates nociceptive neuron firing and causes hyperalgesia in diabetic neuropathy. Nat Med 18: 926–933.
20. Nass N, Vogel K, Hofmann B, Presek P, Silber R-E, et al. (2010) Glycation of PDGF results in decreased biological activity. Int J Biochem Cell Biol 42: 749–754.
21. Bierhaus A, Humpert PM, Morcos M, Wendt T, Chavakis T, et al. (2005) Understanding RAGE, the receptor for advanced glycation end products. J Mol Med 83: 876–886.
22. Neeper M, Schmidt AM, Brett J, Yan SD, Wang F, et al. (1992) Cloning and expression of a cell surface receptor for advanced glycosylation end products of proteins. J Biol Chem 267: 14998–15004.
23. Rabbani N, Thornalley PJ (2012) Methylglyoxal, glyoxalase 1 and the dicarbonyl proteome. Amino Acids 42: 1133–1142.
24. Veiga da-Cunha M, Jacquemin P, Delpierre G, Godfraind C, Théate I, et al. (2006) Increased protein glycation in fructosamine 3-kinase-deficient mice. Biochem J 399: 257–264.
25. Vivacqua A, Lappano R, De Marco P, Sisci D, Aquila S, et al. (2009) G protein-coupled receptor 30 expression is up-regulated by EGF and TGF alpha in estrogen receptor alpha-positive cancer cells. Mol Endocrinol 23: 1815–1826.
26. Roepke TA, Qiu J, Bosch MA, Rønnekleiv OK, Kelly MJ (2009) Cross-talk between membrane-initiated and nuclear-initiated oestrogen signalling in the hypothalamus. J Neuroendocrinol 21: 263–270.
27. Pilkis SJ, Granner DK (1992) Molecular physiology of the regulation of hepatic gluconeogenesis and glycolysis. Annu Rev Physiol 54: 885–909.
28. Thrane S, Lykkesfeldt AE, Larsen MS, Sorensen BS, Yde CW (2013) Estrogen receptor α is the major driving factor for growth in tamoxifen-resistant breast cancer and supported by HER/ERK signaling. Breast Cancer Res Treat 139: 71–80.
29. Schleicher ED, Wagner E, Nerlich AG (1997) Increased accumulation of the glycoxidation product N(epsilon)-(carboxymethyl)lysine in human tissues in diabetes and aging. J Clin Invest 99: 457–468.
30. Oya T, Hattori N, Mizuno Y, Miyata S, Maeda S, et al. (1999) Methylglyoxal modification of protein. Chemical and immunochemical characterization of methylglyoxal-arginine adducts. J Biol Chem 274: 18492–18502.
31. Pourmotabbed T, Creighton DJ (1986) Substrate specificity of bovine liver formaldehyde dehydrogenase. J Biol Chem 261: 14240–14244.
32. Schneider CA, Rasband WS, Eliceiri KW (2012) NIH Image to ImageJ: 25 years of image analysis. Nat Meth 9: 671–675.
33. Nass N, Kukat A, Seibel P, Brömme H-J, Schinzel R, et al. (2009) Advanced glycation end product accumulation in rho$^{(0)}$ cells without a functional respiratory chain. Biol Chem 390: 915–919.
34. Weiner LM, Hu H, Swartz HM (1991) EPR method for the measurement of cellular sulfhydryl groups. FEBS Lett 290: 243–246.

35. Jänicke RU, Sprengart ML, Wati MR, Porter AG (1998) Caspase-3 is required for DNA fragmentation and morphological changes associated with apoptosis. J Biol Chem 273: 9357–9360.
36. Rabbani N, Xue M, Thornalley PJ (2014) Activity, regulation, copy number and function in the glyoxalase system. Biochem Soc Trans 42: 419–424.
37. Toyokuni S, Okamoto K, Yodoi J, Hiai H (1995) Persistent oxidative stress in cancer. FEBS Letters 358: 1–3.
38. Creighton DJ, Zheng Z-B, Holewinski R, Hamilton DS, Eiseman JL (2003) Glyoxalase I inhibitors in cancer chemotherapy. Biochem Soc Trans 31: 1378–1382.
39. Jiang Q, Zheng S, Wang G (2013) Development of new estrogen receptor-targeting therapeutic agents for tamoxifen-resistant breast cancer. Future Med Chem 5: 1023–1035.
40. Droog M, Beelen K, Linn S, Zwart W (2013) Tamoxifen resistance: From bench to bedside. Eur J Pharmacol. 717(1–3):47–57
41. Santen RJ, Fan P, Zhang Z, Bao Y, Song RX-D, et al. (2009) Estrogen signals via an extra-nuclear pathway involving IGF-1R and EGFR in tamoxifen-sensitive and -resistant breast cancer cells. Steroids 74: 586–594.
42. Fukunaga M, Miyata S, Liu BF, Miyazaki H, Hirota Y, et al. (2004) Methylglyoxal induces apoptosis through activation of p38 MAPK in rat Schwann cells. Biochem Biophys Res Commun 320: 689–695.
43. Kuntz S, Kunz C, Rudloff S (2010) Carbonyl compounds methylglyoxal and glyoxal affect interleukin-8 secretion in intestinal cells by superoxide anion generation and activation of MAPK p38. Mol Nutr Food Res 54: 1458–1467.
44. Liu B-F, Miyata S, Hirota Y, Higo S, Miyazaki H, et al. (2003) Methylglyoxal induces apoptosis through activation of p38 mitogen-activated protein kinase in rat mesangial cells. Kidney Int 63: 947–957.
45. Du J, Cai S, Suzuki H, Akhand AA, Ma X, et al. (2003) Involvement of MEKK1/ERK/P21Waf1/Cip1 signal transduction pathway in inhibition of IGF-I-mediated cell growth response by methylglyoxal. J Cell Biochem 88: 1235–1246.
46. Chan W-H, Wu H-J, Shiao N-H (2007) Apoptotic signaling in methylglyoxal-treated human osteoblasts involves oxidative stress, c-Jun N-terminal kinase, caspase-3, and p21-activated kinase 2. Journal of Cellular Biochemistry 100: 1056–1069.
47. Akhand AA, Kato M, Suzuki H, Liu W, Du J, et al. (1999) Carbonyl compounds cross-link cellular proteins and activate protein-tyrosine kinase p60c-Src. J Cell Biochem 72: 1–7.
48. Chang T, Wang R, Olson DJH, Mousseau DD, Ross ARS, et al. (2011) Modification of Akt1 by methylglyoxal promotes the proliferation of vascular smooth muscle cells. FASEB J 25: 1746–1757.
49. Jia X, Chang T, Wilson TW, Wu L (2012) Methylglyoxal mediates adipocyte proliferation by increasing phosphorylation of Akt1. PLoS ONE 7: e36610.
50. Akhand AA, Hossain K, Mitsui H, Kato M, Miyata T, et al. (2001) Glyoxal and methylglyoxal trigger distinct signals for map family kinases and caspase activation in human endothelial cells. Free Radic Biol Med 31: 20–30.
51. Thornalley PJ, Yurek-George A, Argirov OK (2000) Kinetics and mechanism of the reaction of aminoguanidine with the α-oxoaldehydes glyoxal, methylglyoxal, and 3-deoxyglucosone under physiological conditions. Biochemical Pharmacology 60: 55–65.
52. Lykkesfeldt AE, Madsen MW, Briand P (1994) Altered expression of estrogen-regulated genes in a tamoxifen-resistant and ICI 164,384 and ICI 182,780 sensitive human breast cancer cell line, MCF-7/TAMR-1. Cancer Res 54: 1587–1595.
53. Bachmeier BE, Nerlich AG, Rohrbach H, Schleicher ED, Friess U (2008) Maillard products as biomarkers in cancer. Ann N Y Acad Sci 1126: 283–287.
54. Fonseca-Sánchez MA, Rodríguez Cuevas S, Mendoza-Hernández G, Bautista-Piña V, Arechaga Ocampo E, et al. (2012) Breast cancer proteomics reveals a positive correlation between glyoxalase 1 expression and high tumor grade. Int J Oncol 41: 670–680.
55. Germanová A, Germanová A, Tesarová P, Jáchymová M, Zvára K, et al. (2009) Glyoxalase I Glu111Ala polymorphism in patients with breast cancer. Cancer Invest 27: 655–660.
56. Sakamoto H, Mashima T, Sato S, Hashimoto Y, Yamori T, et al. (2001) Selective activation of apoptosis program by S-p-bromobenzylglutathione cyclopentyl diester in glyoxalase I-overexpressing human lung cancer cells. Clin Cancer Res 7: 2513–2518.
57. Ghosh M, Talukdar D, Ghosh S, Bhattacharyya N, Ray M, et al. (2006) In vivo assessment of toxicity and pharmacokinetics of methylglyoxal. Augmentation of the curative effect of methylglyoxal on cancer-bearing mice by ascorbic acid and creatine. Toxicol Appl Pharmacol 212: 45–58.
